# Multi-Voiced Music Bypasses Attentional Limitations in the Brain

**DOI:** 10.3389/fnins.2021.588914

**Published:** 2021-01-27

**Authors:** Karen Chan Barrett, Richard Ashley, Dana L. Strait, Erika Skoe, Charles J. Limb, Nina Kraus

**Affiliations:** ^1^UCSF Sound and Music Perception Lab, Department of Otolaryngology-Head and Neck Surgery, University of California, San Francisco, San Francisco, CA, United States; ^2^Program in Music Theory and Cognition, Bienen School of Music, Northwestern University, Evanston, IL, United States; ^3^Division of Strategy and Finance, Saint Mary’s College, Notre Dame, IN, United States; ^4^Department of Speech, Language, and Hearing Sciences, University of Connecticut, Storrs, CT, United States; ^5^Auditory Neuroscience Laboratory, Department of Communication Sciences and Disorders, Northwestern University, Evanston, IL, United States

**Keywords:** attention, electroencephalography, N100 response, multivoiced music, counterpoint, polyphony, auditory scene analysis

## Abstract

Attentional limits make it difficult to comprehend concurrent speech streams. However, multiple musical streams are processed comparatively easily. Coherence may be a key difference between music and stimuli like speech, which does not rely on the integration of multiple streams for comprehension. The musical organization between melodies in a composition may provide a cognitive scaffold to overcome attentional limitations when perceiving multiple lines of music concurrently. We investigated how listeners attend to multi–voiced music, examining biological indices associated with processing structured versus unstructured music. We predicted that musical structure provides coherence across distinct musical lines, allowing listeners to attend to simultaneous melodies, and that a lack of organization causes simultaneous melodies to be heard as separate streams. Musician participants attended to melodies in a Coherent music condition featuring flute duets and a Jumbled condition where those duets were manipulated to eliminate coherence between the parts. Auditory–evoked cortical potentials were collected to a tone probe. Analysis focused on the N100 response which is primarily generated within the auditory cortex and is larger for attended versus ignored stimuli. Results suggest that participants did not attend to one line over the other when listening to Coherent music, instead perceptually integrating the streams. Yet, for the Jumbled music, effects indicate that participants attended to one line while ignoring the other, abandoning their integration. Our findings lend support for the theory that musical organization aids attention when perceiving multi–voiced music.

## Introduction

Humans are limited in their ability to perform two things at once as the performance of simultaneous tasks results in task interference (Neisser and Becklen, 1975; Pashler, 1992; Simons and Chabris, 1999; Herath, 2001; Simons, 2010). Attention is a central bottleneck (Pashler, 1994). Within the auditory domain, it is difficult-to-impossible to attend to multiple independent auditory events simultaneously (Eramudugolla et al., 2005). The “cocktail party problem” refers to the difficulty of paying attention to a single conversation within a crowded room; humans can only pay close attention to one conversation, not several (Cherry, 1953; Hayken et al., 2005).

Speech and music are both complex acoustic stimuli but are fundamentally different. With speech, streams (e.g., a single voice) are heard independently, rarely relying on their integration to convey meaning. Empirical studies of verbal interactions have uncovered certain basic rules of human communication. Within conversations, speakers take turns, leaving little temporal gaps in between turns of speakers, and the overlap between speakers is brief (Sacks et al., 1974; Wooffitt, 2005). There is a relatively orderly process by which conversations unfold (Schegloff, 1987, 1996, 2007). With two people speaking simultaneously, the speakers utterances are not cooperative, but competitive, violating rules of effective communication. With music, however, simultaneous melodies can be both independent and interdependent: possessing their own character (independence), yet also blending to create the composite structure (interdependence). Music and conversation differ markedly in their relative structures. Because of this, the organization of music – its structure – may be one of the key differences between music and speech.

Factors like timbre, rhythm and register aid in the separation of concurrent lines, in what is termed auditory scene analysis (Wright and Bregman, 1987; Bregman, 1990). Comprehending music, however, demands both an appreciation of how individual melodies fit together as well as tracking them individually, a cognitive balance between auditory segregation and integration (Keller, 2008; Ragert et al., 2014; Disbergen et al., 2018). This is particularly true of Baroque polyphony: complex, multi-voiced music written in the 17th and 18th centuries. But how is such polyphony comprehended, given known limitations in human attention?

Experiments investigating attention in music listening have produced opposing theories to explain the perception of polyphony. Gregory (1990) proposed a “divided attention model,” which suggested that listeners are capable of simultaneously perceiving multiple musical streams. Sloboda and Edworthy (1981) proposed a different model of attention – the “figure-ground model.” In this model, listeners focus on one melody while staying aware of the melody temporarily relegated to the background. By shifting concentration onto different parts, the perception of the music changes; both parts of the percept are processed, but the “figure” or foreground melody receives a different processing compared to the “background.” Bigand et al. (2000) contrasted (a) an “Integrative Model of Attention,” where listeners integrate two or more voices of polyphony into a single stream, and (b) another model with listeners rapidly shifting their attention between musical lines. This latter attention model, otherwise known as “attentional switching,” allows attention to rove between the two melodies, alternately paying attention to one line and then the other.

Multi-voiced music therefore provides an intriguing domain to examine attention and how the auditory system copes with multiple independent inputs (Madsen, 1997; Bigand et al., 2000; Keller, 2001; Ragert et al., 2014). Composers organize the music carefully to highlight individual lines while also promoting a holistic gestalt that integrates multiple lines. Musical structure, or the coherence it yields, may be the prime framework allowing listeners to comprehend multiple melodies in a way that is impossible with multiple independent speech streams.

Electroencephalography (EEG) has emerged as a strategy for studying how listeners parse complex soundscapes. One popular paradigm, the dichotic listening paradigm, asks participants to listen to two simultaneous auditory streams, one directed to each ear. Participants are told to attend to one stream while ignoring the other. Auditory cortical-evoked responses are collected to attended and ignored probes buried within these streams. The N100 response (also known as “N1”) is a negative cortical component occurring roughly 100 ms after probe onset. Importantly, the N100 response has been widely used as a neural index of attention: N100 amplitude has been found to be more negative to attended than ignored stimuli (Hillyard et al., 1973), a phenomenon referred to here as the “N100 attention effect.” The dichotic paradigm has been employed with simple tone patterns (Snyder et al., 2006) and concurrent speech streams (Coch et al., 2005; Sanders et al., 2006; Stevens et al., 2006, 2009).

**FIGURE 1:**
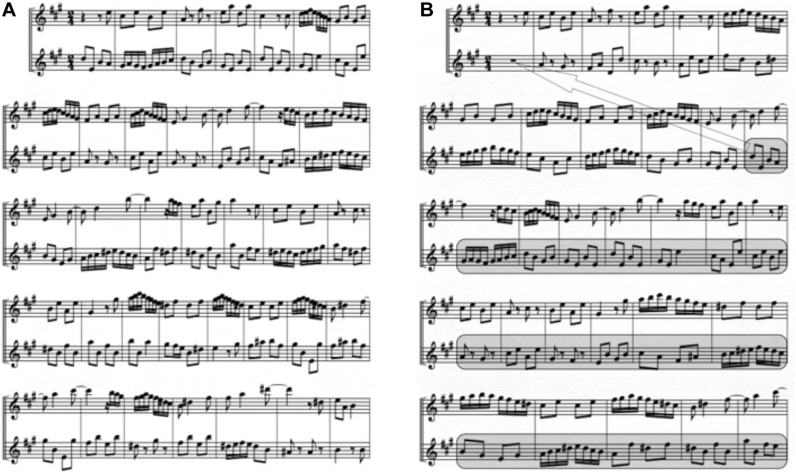
Sample portion of Musical Excerpts. **(A)** shows sample of Coherent excerpt. Music in Coherent excerpts were presented as written by the composers. **(B)** shows sample of corresponding Jumbled excerpt. The score was scrambled and pulled apart by taking large portions of one line (see highlighted box for example) and inserting it in a different location. Additionally, the *tempo* (i.e., speed) of bottom line was slower than *tempo* of top line. Coherence between the two lines has been destroyed, although each line sounded relatively intact when played alone because chunks were excerpted at natural stopping points (ex. cadences or other structural pauses). Excerpt sounds as if the two melodies are playing completely independently and separately.

Prior experiments have investigated the neural networks underlying perception of multi-voiced music (Satoh et al., 2001; Janata et al., 2002; Uhlig et al., 2013). To our knowledge, however, no experiment has specifically adapted the dichotic listening paradigm to study neural indices of attention when listening to real-world melodies. It was hypothesized that: (1) musical structure provides coherence across different musical lines, creating a cognitive framework for listeners to attend to co-occurring melodies and that (2) lack of coherence-creating structure would cause melodies to be heard as separate streams rather than as an integrated percept. To test these hypotheses, two musical conditions were created: (1) a *Coherent* music condition using Baroque counterpoint duets and (2) a *Jumbled* condition in which the lines of the duets were scrambled, and the melodies played asynchronously with one another, no longer forming coherent compositions (Figure 1). In both conditions, listeners were instructed to selectively attend to one melody while ignoring the other. By using the dichotic listening paradigm, this experiment examined how musical structure affects attention to multi-voiced music at a biological level in trained musician participants. Since we propose that musical structure facilitates attention to multiple melodies, we hypothesized that the two musical conditions would elicit different “N100 attention effects.” It was predicted that smaller (if any) N100 attention effects would be seen for the normal Coherent music condition compared to the Jumbled. In normal music, melodies in a composition are meant to be integrated into a single gestalt, and this, we argue, strongly interferes with the ability to selectively attend to one stream and ignore the other, hence small if any, N100 selective attention effects will be seen. By contrast, the Jumbled condition is predicted to yield larger N100 attention effects, because the broken coherence helps to focus attention onto a single stream. In this cacophonous Jumbled music, it is relatively easy to selectively attend to one line while ignoring the other since the two lines are not meant to sound like a cohesive unit. By testing trained musicians well-versed in navigating the attentional demands of multipart musical structures, this experiment aims to illuminate the biological, attentional mechanisms that support the comprehension of sophisticated musical soundscapes. Studying expert listeners may shed light on how and why humans can perceive polyphony – a process that should be challenging and yet occurs relatively effortlessly among listeners from a variety of musical cultures.

## Methods

### Participants

All experimental procedures were approved by the Northwestern University Institutional Review Board. 21 young adult musicians (11 females), ages 18–37 (mean = 23.81 ± S.D 4.80 years) recruited from Northwestern University participated in this study. All participants had normal hearing (<20 dB HL pure tone thresholds at octave frequencies from 125 to 8000 Hz), no reported history of neurological or learning disorders, and normal IQ (mean = 125.76 ± SD 9.71) as measured by the 2-subtest Wechsler Abbreviated Scale of Intelligence (WASI) (Harcourt Assessment, San Antonio, TX, United States) thereby passing our screening procedures. Participants were all actively practicing their instrument at time of testing and had a mean of 12 years of musical training (SD = 5.22 years). For this experiment, only highly trained-musicians were tested because pilot testing revealed that the paradigm was challenging (see Methods below). Oboists and clarinetists were excluded from participating as the sounds of those instruments were used in the study, and previous research has found that musicians show enhanced neural activity and preferential attention to the sound of their instrument (Pantev et al., 2001a; Shahin et al., 2003, 2008; Margulis et al., 2007; Strait et al., 2012). All participants had significant small ensemble experience or played a multi-line instrument, ensuring familiarity with hearing multi-voice music.

### Musical Stimuli

There were two experimental music conditions: Coherent and Jumbled. Stimuli consisted of four excerpts from Johann Philippe Quantz’s *Six Duets for Two Flutes*, Op. 2 and George Telemann’s *Six Sonatas for Two Flutes*, TWV 40, No. 103 and 107. The Coherent music condition featured the compositions as written by the composers. In the Jumbled condition, one line was played at a slower *tempo* than the other and the score was pulled apart and subsequently rearranged so that musical figures in one line no longer coordinated with those in the other line. Thus, in the Jumbled condition the music lacked structural coherence *between* the two lines because of the scrambling of the music (see Figure 1 for details).

All musical excerpts were transcribed into Sibelius 4 (Sibelius, Daly City, CA, United States) and MIDI files were exported into Logic Pro 8 (Apple Inc., Cupertino, CA, United States). Stock oboe and bass clarinet sounds were replaced with high-quality, realistic-sounding instruments from the EastWest Sound Library (EastWest Studios/Quantum Leap, Hollywood, CA, United States). Note that this resulted in two lines which were in different timbres and registers. Although the Baroque duets were written for two flutes, instruments and registers were altered here; pilot testing revealed that selective attention to one line was too difficult even for expert listeners when melodies were played by the same instrument in the same register. To ensure and verify that participants were paying attention during the experiment, a behavioral, perceptual task was given. Since it was difficult to ask concrete questions about the music (i.e., simple questions easily yielding objective answers), participants were told to count “target tones” – infrequently occurring, quarter-tone flat mistunings inserted into the line. Versions of sound files were made where the intended “attended” line featured 3–7 randomly chosen, quarter-tone flat mistunings during each 3-min excerpt. These target tone mistunings were made using the Pitch Bend Plugin in Logic Pro 8. At the end of the excerpt, participants were quizzed on the total number of mistunings detected as a screening method for ensuring they were paying attention and following experimental directions (see Methods below). The melodies were normalized for volume using the Level16 sound editing program (Tom Carrell and Bob Tice, University of Nebraska).

### Electrophysiology

#### N100 Evoking (Probe) Stimulus

The evoking stimulus was a Steinway piano sound (G_1_, F0 = 100 Hz, 200 ms) synthesized using Logic Pro (Apple Inc) that was superimposed into the musical lines, occurring at randomized interstimulus intervals (ISIs) of 600, 900, or 1200 ms. This sound possesses a sharp onset that is conducive to recording robust N100 responses.

#### Recording Parameters and Protocol

Auditory-evoked potentials were recorded to the probe using a 32-channel silver electrode cap (Electrocap International, Eaton, OH, United States) in NeuroScan Acquire 4.3 (Compumedics) while participants were seated in a sound-attenuated booth. A single electrode was placed on each of the right and left earlobes; right ear acted as reference during the online recording and the recordings were re-referenced to linked earlobes offline. Single electrodes were placed on the medial canthus of the right eye and on the lower eyelid of the left eye to act as eye-blink monitors, so that trials containing eyeblink artifacts could be rejected from the average. Contact impedance for all electrodes was under 10 kΩ (Ferree et al., 2001; Kappenman and Luck, 2010). Neural recordings were off-line filtered from 0.1 to 100 Hz and digitally sampled at a rate of 500 Hz.

The probe was presented with the contrapuntal melodies played through two wall-mounted speakers located exactly 1 meter to the left and right of the participant at 180° apart from one another. The melodies were played dichotically, one to each speaker. Participants were asked to attend to one of the two simultaneously presented melodies which differed in location (left/right speaker), instrument (bass clarinet or oboe), and musical content. This procedure was adapted from previous experiments that used dichotic speech streams (Coch et al., 2005; Sanders et al., 2006; Stevens et al., 2009). Participants were initially told which instrument to attend to and were directed to which speaker the instrument would be presented from. The attended instrument and its initial location (right/left) were randomized across participants, as was the order of the Jumbled and Coherent conditions. The probe was presented randomly to the left or right (i.e., attended or ignored) sides of the head. The musical melodies were played at 55 dB SPL and the probe was played at 65 dB SPL, creating a 10 dB difference in keeping with protocol used in other dichotic listening paradigms (Coch et al., 2005; Sanders et al., 2006; Stevens et al., 2009). The recording took place in four three-minute blocks. After each 3-min block, participants were quizzed on the number of mistunings they heard in the attended melody to ensure active engagement and listening. To control for any ear advantages, the melody then switched sides and participants were asked to change their attended side (left/right) in order to continue the task with the same melody and timbre. For example, if the bass clarinet melody originally came out of the left speaker, in the next 3-min segment it originated from the right speaker. Participants were told to switch their attention to the other speaker.

All participants were able to perform the selective attention task as indicated by their performance in tracking the number of target tone mistunings in the attended line (average percent correct = 80%).

### Data Processing and Analysis

The continuous recordings were bandpass filtered offline from 0.1 to 40 Hz (12 dB/octave, zero phase shift) and subjected to a spatial filtering algorithm in Neuroscan Edit 4.3 (Compumedics) to reduce the influence eye blinks on the recordings. For each musical condition, recordings were epoched over a window of −100 to 500 ms, using the onset of the probe stimulus to define 0 ms. Epochs containing muscle artifact that exceeded a ± 100 μV threshold were removed using a spatial algorithm where the algorithm computes the degree of similarity between each epoch and the average of all epochs using Pearson’s correlations. Individual responses were ranked according to their Pearson’s *r*-values and the most poorly correlated 30% were discarded. The remaining 70% were averaged, making up the final averaged evoked response for each subject in each condition (Abrams et al., 2008); these 500 artifact free responses from each participant were subsequently used for statistical analysis.

#### Statistical Analysis

**FIGURE 2:**
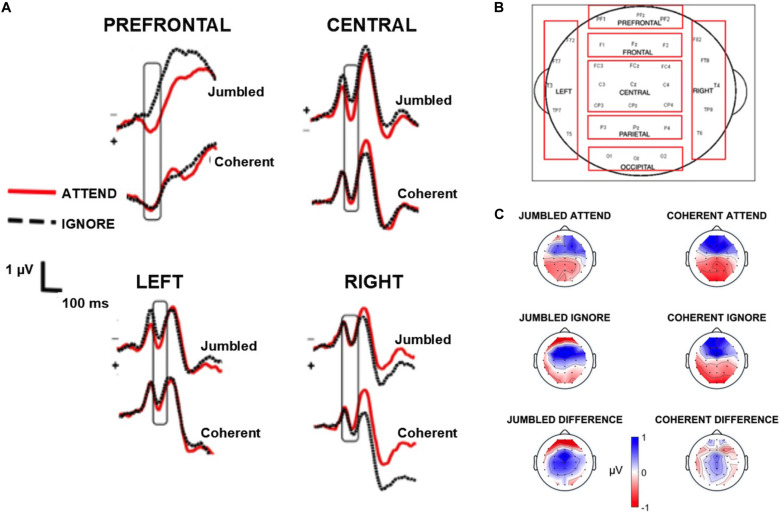
EEG waveforms. **(A)** shows the grand average waveforms highlighting the N100 component (box) for the four regions of interest [prefrontal, central, left, and right regions, see **(B)**]. **(C)** depicts the topographic maps of the N100 component for the four conditions as well as the difference between attend and ignore conditions, generated by subtracting the topographic map of the attend from the ignore conditions. Topographic maps zoom in at 115 ms where the greatest negativity occurs in the grand average waveforms.

Mean amplitudes for the N100 cortical response (occurring from 100 to 150 ms following stimulus onset) were calculated for each electrode channel. This time range aligns with well-described characteristics of the N100. Mean Amplitudes during this time window were then averaged into scalp regions of interest (ROI, see Figure 2) by mathematically averaging the mean amplitudes from individual channels; these ROI blocks were based on precedent set from a previous paper (Strait et al., 2014). This resulted in a single amplitude for each ROI for each participant in both the attend and ignore conditions. Visual inspection of waveforms revealed that responses at the Frontal, Parietal, and Occipital ROI were not robust (i.e., did not resemble a clean auditory-evoked response) and therefore excluded from analysis. Differences in N100 amplitudes were compared across musical conditions using a 2 × 2 × 4 RMANOVA with musical condition (Jumbled vs. Coherent), attention (Attend vs. Ignore), and scalp ROI (Prefrontal, Central, Left, Right, see Figure 2) as within-subject factors. Data was normally distributed as assessed by the Kolmogorov Smirnov test of normality. Mauchly’s Test of Sphericity showed that the assumption of sphericity was violated, therefore RMANOVA statistics are reported below using the Greenhouse-Geisser correction (see Results).

## Results

### Summary of Results

Participants demonstrated significant selective attention effects in the Jumbled Condition but not the Coherent Condition, particularly in prefrontal and central scalp regions (see below and Table 1).

**TABLE 1:** Within-music comparisons for N100 amplitudes.

Scalp region	JUMBLED Attend vs. Ignore	COHERENT Attend vs. Ignore
Prefrontal	*t* = −2.13, *p* = 0.046*	*t* = **−**0.029, *p* = 0.98
Central	*t* = −2.45, *p* = 0.024*	*t* = **−**0.32, *p* = 0.75
Left	*t* = **−**0.273, *p* = 0.79	*t* = **−**1.58, *p* = 0.13
Right	*t* = 1.12, *p* = 0.28	*t* = 0.32, *p* = 0.75

Table shows *post hoc* paired *t*-tests comparing Attend to Ignore N100 amplitudes over each scalp region of interest and within each musical condition. The Jumbled condition shows significant N100 attention effects (i.e., significantly more negative Attend amplitudes compared to Ignore amplitudes) in prefrontal and central sites (see bolded values), while the Coherent music condition shows no effect of attention *p < 0.05.

### N100 Attention Effect Seen in Jumbled Condition but Not Coherent Condition

Analysis revealed a non-significant but trending effect of attention *F*(1,20) = 3.49, *p* = 0.077 and a significant main effect of condition *F*(1,20) = 5.23, *p* = 0.033. Non-significant but trending two-way interaction effects were seen both (1) between attention and condition *F*(1,20) = 3.39, *p* = 0.080, suggesting that attention might be impacting the two musical conditions differently, and (2) between condition and scalp region *F*(1.05,21) = 3.47, *p* = 0.075 suggesting that the cortical regions were eliciting different responses in the two musical conditions. Finally, a non-significant but trending three-way interaction between attention, condition, and scalp region was observed *F*(1.32,26.35) = 3.06, *p* = 0.082.

Following a main effect of condition as well as trending interaction effects, *post hoc* paired sample *t*-tests were employed comparing Attend vs. Ignore amplitudes within each musical condition (Table 1).

**FIGURE 3:**
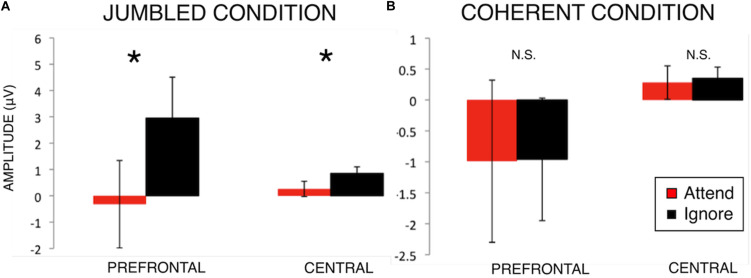
N100 amplitudes for Attend and Ignore responses in the Jumbled Condition **(A)** and Coherent Condition **(B)**. Bar graphs comparing N100 group-mean amplitudes in both music conditions (mean ± S.E.) for Prefrontal and Central scalp regions of interest. An effect of attention on the N100 response magnitude (i.e., N100 attention effect) is seen in the Jumbled music condition (cacophonous compositions, **A**) but not the Coherent music condition (normal music compositions, **B**). *p < 0.05.

*Post hoc* paired-sample *t*-tests were performed comparing Attend to Ignore (Table 1 and Figure 3). In the Jumbled condition, Attend amplitudes were significantly more negative than Ignore amplitudes at the Prefrontal region [*t*(20) = −2.13, *p* = 0.046] and Central region [*t*(20) = −2.45, *p* = 0.024]. Thus, in the Jumbled condition a selective “N100 attention effect” was seen; the amplitude of the N100 response was more negative to attended rather than ignored stimuli (Figure 3). This suggests that participants were able to selectively attend to one line over another. No significant differences between Attend and Ignore amplitudes, however, were found for the Coherent music condition (Figure 3).

## Discussion

The effect of musical structure on attention was examined to evaluate the hypothesis that musical coherence aids the processing of simultaneous musical melodies. This hypothesis was investigated through neural indices of attention when musicians listened to incoherent versus coherent musical stimuli. Results showed that attentional responses are modulated by whether the musical lines were coherent or disjunct: (1) No N100 selective attention effect was found when participants heard normally structured polyphonic compositions (Coherent condition) in that the attend and ignore conditions did not differ from each other; (2) An N100 selective attention effect, however, was found when listening to incoherent music derived from the coherent excerpts (Jumbled condition). These results suggest that musician participants were capable of selectively attending to one line while ignoring the other when the lines no longer blended together to form an intact composition. When listening to coherent music, however, they integrated the two melodies into one percept.

These results provide biological support for the “integrative model of attention” that suggested that listeners integrate two or more voices of polyphony into a single perceptual stream (Bigand et al., 2000). Our results cannot rule out the “selective attention model” for the Coherent condition as it is possible that participants’ attention flickered between streams so rapidly as to not affect the N100 cortical response. While possible, the results, especially the fact that Ignored inputs were similar to Attend inputs when listening to coherent music (Table 1), seem to more plausibly support an “integrative model of attention.” Note that the Jumbled music condition with incoherent musical lines supported the selective attention model. Taken together, results support our hypothesis that musical organization facilitates attention to multi-voiced music. The Coherent musical condition resulted in listeners using integrative listening strategies while the Jumbled condition yielded selective attention effects. Musical structure may compensate for attentional limitations typically experienced when two auditory signals are presented concurrently.

Here, “musical structure” loosely describes coherence, or the global organization of a composition. The key difference between the Coherent and Jumbled music conditions is that the melodies in the Jumbled condition eliminated correspondence between the two lines and they moved asynchronously with each other, disrupting meter and tonality. Periodic rhythmic structures and metric frameworks may play a key part in attention and integrative listening to music (Keller, 2001; Hurley et al., 2018), freeing attentional resources to efficiently process multiple parts (Keller, 1999; Keller and Burnham, 2005). Furthermore, factors like unstable tonality have been predicted to interfere with integrative listening as well (Keller, 2001). These ideas support the concept that in the Jumbled music condition, by virtue of its disorganization, it was difficult to fuse the two lines.

This “disorganization” may account for why the Jumbled music condition yielded N100 results similar to those found in simultaneous speech conditions used in other studies (Coch et al., 2005; Sanders et al., 2006; Stevens et al., 2009). A parallel exists between competing conversational speech effects, where two people are talking over each other, and the cacophonous music used in the Jumbled music condition. The organization underlying “normal” music may be the vital distinguishing feature that sets music apart from other auditory inputs like speech. Multiple speakers are heard as competing when they talk at the same time, but simultaneous melodies are composed to reinforce and complement one another.

To our knowledge, this is the first study using naturalistic musical materials in a dichotic listening paradigm to investigate neural indices of attention with concurrent musical lines. Even so, the polyphonic duets used featured relatively simple counterpoint. Future experiments may want to employ more complex polyphonic compositions (e.g., fugues, Baroque trios). Additional information about the neural activation patterns behind segregation and integration may be gained through the incorporation of neural imaging techniques. It is thought that the planum temporale (PT) is involved in stream segregation while the inferior parietal cortex (IPC) is involved in auditory integration (see Gutschalk and Dykstra, 2014; Ragert et al., 2014).

In this experiment, only the effect of musical structure, an exogenous factor of attention, was investigated when listening. Different types of musical training, conceivably considered as endogenous factors of attention, may also influence indices of attention when perceiving polyphony. For example, adult musicians demonstrate enhanced cortical and subcortical responses to the timbre of their own instruments, showing a preference for their major instrument (Pantev et al., 2001b; Margulis et al., 2007; Shahin et al., 2008; Trainor et al., 2009; Strait et al., 2012; Barrett et al., 2013; Shahin et al., 2003). This experiment specifically excluded musicians who played oboe or clarinet, the instruments used in the stimuli, to control for any possible timbre preference. Future experiments may want to investigate the interaction between endogenous, top-down (extramusical or training-related) and exogenous, bottom-up (music structure-related) factors that affect attention. Would musicians’ preference for their own timbre override or change their responses to the musical structure and its effect on attention?

Aside from examining musicians playing different instruments, one could also divide subject groups according to various aspects of musical training. Participants here were experienced in listening to simultaneous musical lines. Future research might investigate whether neural responses differ between instrumentalists who play single-line instruments (ex. flute, oboe) as opposed to those who play multi-line instruments (e.g., organ, piano, keyboard instruments). Additionally, neural responses might differ between musicians with minimal vs. substantial amounts of aural skills training since ear training aims to develop an ability to hear multiple concurrent lines. Moreover, non-musicians and those struggling with acoustic scene analysis, such as cochlear implant users who receive degraded auditory input, may show different results as well.

In conclusion, this experiment demonstrated that musical organization facilitates attention to the broader musical context when trained musicians listened to multi-voiced music as evidenced through auditory cortical-evoked potentials. Musical structure may help humans process simultaneous melodies as a way to cope with the attentional limitations that one would have for other auditory stimuli (such as speech). This organization may allow listeners to integrate musical melodies into one percept, thereby aiding in the comprehension of polyphonic music.

## Data Availability Statement

The raw data supporting the conclusions of this article will be made available by the authors, without undue reservation.

## Ethics Statement

The studies involving human participants were reviewed and approved by the Northwestern University Institutional Review Board. The patients/participants provided their written informed consent to participate in this study.

## Author Contributions

KB designed the experiment, collected and analyzed the data, wrote the manuscript with feedback from co-authors. RA advised KB in design of the experiment and contextualization of scientific work. DS co-designed the experiment, aided in technical support in EEG collection, and co-analyzed the data. ES wrote the scripts that allowed for data analysis and aided in making figures. CL co-wrote the manuscript. NK oversaw the entire experiment from experimental design to data collection to manuscript preparation. All authors contributed to the article and approved the submitted version.

## Conflict of Interest

The authors declare that the research was conducted in the absence of any commercial or financial relationships that could be construed as a potential conflict of interest.
